# Supplementary material for: SARS-CoV-2 Infection in Cities from the Southern Region of Bahia State, Brazil: Analysis of Variables Associated in Both Individual and Community Level
Source: Viruses. 2023 Jul 20;15(7):1583. doi: 10.3390/v15071583 (PMC10383252; doi:10.3390/v15071583)
Supplement: Supplementary file 1 [file viruses-15-01583-s001.zip › viruses-2514525-supplementary.pdf]

viruses

MDPI

Supplementary Material

SARS-CoV-2 Infection in Cities from the Southern Region of Bahia State, Brazil: Analysis of Variables Associated in Both Individual and Community Level

Table S1. Geographical location of cities from Southern Region of Bahia State includes in the study.

ID	City	Geographical Location	Population[a]	Total Samples
1	Almadina	14° 42′ 18″ S - 39° 38′ 13″ W	6.357	43
2	Arataca	15º 15' 48'' S - 39º 24' 52'' W	10.392	130
3	Barro Preto	14° 47′ 34″ S - 39° 27′ 58″ W	6.453	4
4	Belo Campo	11° 33' 0'' S - 41° 37' 0'' W	16.021	1
5	Buerarema	14° 56′ 50″ S - 39° 18′ 16″ W	18.605	651
6	Camacan	15° 24′ 51″ S - 39° 30′ 4″ W	31.472	358
7	Camamu	13° 56′ 29″ S - 39° 7′ 17″ W	35.180	1
8	Coaraci	14° 38' 29'' S - 39° 33' 1'' W	20.964	5
9	Floresta Azul	14° 50' 52'' S - 39° 39' 23'' W	10.660	585
10	Gandu	13° 44′ 18″ - 39° 28′ 49″ W	30.336	1
11	Ibicaraí	14° 50′ 29″ S - 39° 35′ 34″ W	24.272	8
12	Ibicuí	14° 49' 48'' S - 39° 58' 58'' W	15.785	2
13	Ilhéus	14° 47′ 50″ S - 39° 2′ 8″ W	184.236	287
14	Itabuna	14° 47′ 21″ S - 39° 16′ 40″ W	204.667	131
15	Itaju do Colônia	15° 8′ 34″ S - 39° 43′ 35″ W	7.309	1
16	Itajuípe	14° 41′ 7″ S - 39° 21′ 52″ W	21.081	1108
17	Itaparica	12° 55′ 2″ S - 38° 39′ 17″ W	20.725	1
18	Itapé	14° 52′ 35″ S - 39° 25′ 59″ W	10.995	616
19	Itapetinga	15° 15′ 23″ S - 40° 15′ 27″ W	68.273	2
20	Itapitanga	14° 24′ 58″ S - 39° 32′ 45″ W	10.207	222
21	Itororó	15° 7′ 3″ S - 40° 4′ 5″ W	19.914	3
22	Ituberá	13° 43′ 57″ S - 39° 9′ 2″ W	26.591	2
23	Jussari	15° 10' 10'' S - 39° 29' 18'' W	6.474	259
24	Maraú	15° 10' 10'' S - 39° 29' 18'' W	19.101	2
25	Mascote	15°33'47.02"S - 39°18'9"W	14.640	2
26	Pau Brasil	15°27'51.01"S - 39°39'4"W	10.852	524
27	Presidente Tancredo Neves	13° 27′ 14″ S - 39° 25′ 15″ W	23.845	2
28	Salvador	12° 58′ 13″ S - 38° 30′ 45″ W	2.675.656	2
29	Santa Cruz da Vitória	14° 57′ 42″ S - 39° 48′ 37″ W	6.673	1
30	Santa Luzia	10° 58′ 60″ S - 45° 33′ 0″ W	13.344	5
31	Ubaitaba	14° 18′ 17″ S - 39° 19′ 24″ W	20.691	3
32	Una	15° 16′ 11″ S - 39° 4′ 10″ W	24.110	4
33	Uruçuca	14° 35′ 12″ S - 39° 17′ 29″ W	19.837	3
34	Vitória da Conquista	14° 51′ 53″ S - 40° 50′ 13″ W	306.866	1

[a] Population according to Instituto Brasileiro de Geografia e Estatística (IBGE) census 2010 (www.ibge.gov.br accessed on 16 June 2023).

Table S2. *P* values of the Pearson correlation matrix.

	D_i	P_i	HDI_c	AWS_c	$SR_{6\text{-}14}$
D_i	-	0.0935	0.0442	0.0137	0.6584
P_i	0.0935	-	0.0339	0.8680	0.5176
HDI_c	0.0442	0.0339	-	0.3174	0.5113
AWS_c	0.0137	0.8680	0.3174	-	0.5242
$SR_{6\text{-}14}$	0.6584	0.5176	0.5113	0.5242	-

D_i, Demand index; P_i, Positivity index; HDI_c, Human development index; AWS_c, Average worker salary; $SR_{6\text{-}14}$, Schooling rate between 6-14 years-old.
